# CX3CL1(+) Microparticles-Induced MFG-E8 Enhances Apoptotic Cell Clearance by Alveolar Macrophages

**DOI:** 10.3390/cells10102583

**Published:** 2021-09-28

**Authors:** Wen-Hui Tsai, Shao-Chi Chang, Yu-Chieh Lin, Hui-Chi Hsu

**Affiliations:** 1 Department of Respiratory Therapy, Taipei Medical University, Taipei 106, Taiwan; tsaiwh@gmail.com; 2 Department of Physiology, School of Medicine, National Yang-Ming Chiao-Tung University, Taipei 112, Taiwan; cha108001@chgh.org.tw (S.-C.C.); b117094033@gmail.com (Y.-C.L.); 3 Sleep Medicine Center, Division of Chest Medicine, Taichung Tzu Chi Hospital, Taichung 427, Taiwan; 4 Department of Medicine, School of Medicine, National Yang-Ming Chiao-Tung University, Taipei 112, Taiwan; 5 Division of General Medicine, Department of Medicine, Taipei Veterans General Hospital, Taipei 112, Taiwan; 6 Division of Hematology & Oncology, Department of Medicine, Chan-Hsin General Hospital, Taipei 112, Taiwan

**Keywords:** CX3CL1, milk fat globule-epidermal growth factor 8 (MFG-E8), microparticles, phagocytosis, resolution, apoptosis, acute lung injury

## Abstract

During the resolution phase of acute lung injury, apoptotic cells release CX3CL1 as a “find-me” signal to attract alveolar macrophage transmigration toward apoptotic cells for phagocytosis. However, it is still not clear whether CX3CL1 has pro-phagocytic activity on alveolar macrophage. In this study, we investigated the role of apoptotic NB4 cells-derived CX3CL1(+) microparticles (apo-MP) on the phagocytic activity of NR8383 cells. We demonstrate that exogenous CX3CL1 and apo-MP enhanced the phagocytic activity of NR8383 cells in a CX3 CR1-dependent manner. The apo-MP-enhanced phagocytic activity on NR8383 was attenuated when apo-MP and NR8383 cells were pre-treated with anti-CX3CL1 antibodies and anti-CX3CR1 antibody, respectively, before incubating both for phagocytic assay. Further studies demonstrate that exogenous CX3CL1 and apo-MP also enhanced NR8383 cells in their surface expression and release of MFG-E8 in a CX3CR1 dependent manner. The enhanced phagocytic activity of CX3CL1-treated NR8383 cells was attenuated when NR8383 cells were pre-treated with an anti-MFG-E8 antibody before CX3CL1 treatment. We conclude that apoptotic cell-derived CX3CL1(+) microparticles enhance the phagocytic activity of NR8383 cells by up-regulating their MFG-E8 as a bridge molecule, and these contribute to the formation of phagocytic synapses between apoptotic cells and alveolar macrophages for the subsequent phagocytic clearance of apoptotic cells.

## 1. Introduction

All-trans retinoic acid (ATRA) is the standard of care in the treatment of acute promyelocytic leukemia (APL) by inducing APL cell differentiation into mature granulocytes. However, this treatment can be complicated by the occurrence of differentiation syndrome (DS; also known as retinoic acid syndrome or cytokine storm) in up to 25% of treated patients [1]. DS has the full clinical manifestations of acute lung injury, including fever, dyspnea, hypoxemia, diffuse pulmonary infiltrate detectable on chest X-ray, and pleural or pericardial effusion [1,2,3,4]. In vitro studies have suggested that ATRA acts through the aberrant retinoid receptor PML-RARA in APL cells to induce granulocytic differentiation, and this is also associated with (a) increased production of many inflammatory vasoactive cytokines, including interleukin (IL)−1, IL−6, IL−8, monocyte chemotactic protein−1 (MCP−1), tumor necrosis factor alpha (TNF-α), and CCL2 [5,6,7]; (b) an excess release of cathepsin G to enhance capillary permeability and damage [8]; and (c) upregulation of leukocyte integrins to promote their adherence to capillary endothelium and organ infiltration [1,6,9]. These events lead to massive ATRA-APL cells transmigration from blood vessels into alveolar spaces and cause acute lung injury in DS patients.

In the early inflammation phase of acute lung injury, alveolar macrophages (AM) strategically located in the alveolar space destroy invading pathogens including microorganisms and environmental toxins, while numerous neutrophils are rapidly recruited from the bloodstream to the alveolar space [10]. Coordinated resolution programs initiate shortly after the beginning of inflammatory responses to create a favorable environment for the resolution phase to take place. Upon entering alveolar spaces, the activated neutrophils release microparticles (MP) containing pro-resolution mediators (e.g., annexin A1) to control further granulocyte ingress and turn on the resolution programs in alveolar spaces [11,12,13,14], while many resolution mediators engage in activating apoptosis among neutrophils by down-regulating their survival pathways [15]. Apoptotic neutrophils release pro-resolution mediators such as annexin A1 to promote monocyte recruitment into alveolar spaces while inhibiting neutrophil transmigration [16]. Attenuation of the effects of pro-inflammatory mediators assists in the successful ‘switching off’ of inflammation when sufficient numbers of cells have been recruited into alveolar spaces [17]. Regarding this, chemokine proteolysis, sequestration by atypical receptors, and degradation by neutrophil extracellular traps are important mechanisms to build up chemokine gradients in restricting the influx of neutrophils. In addition, inflammatory mediators may also induce a negative-feedback loop by down-regulating the production of pro-inflammatory cytokines [18].

In the resolution phase of acute lung injury, the phagocytic clearance of apoptotic cells by AM, also referred to as efferocytosis, plays the crucial role of bringing the alveolar space back to its normal function [19]. Efferocytic engulfment of dying cells by macrophages requires the formation of the phagocytic synapse between apoptotic cells and phagocytes, and these are regulated by a network of “find-me”, “eat-me”, and “don’t eat me” signals; bridging molecules; and specialized phagocytic receptors on the surface of both cells [20,21,22], through which apoptotic neutrophils promote their own clearance by expressing “find-me” and “eat-me” signals to attract macrophage transmigration and allow the identification of the dying cell. CX3CL1, also known as fractalkine, is released by apoptotic cells and acts as a “find-me” signal, which binds to CX3 C-motif chemokine receptor 1 (CX3CR1) on macrophages to attract their migration toward apoptotic cells along the chemotactic gradient of CX3CL1 [23]. Recently, we have reported that CX3CL1 and CX3CL1(+) MP released by apoptotic ATRA-APL cells (apo-MP) attract AM migration toward apoptotic ATRA-APL cells [24,25]. However, whether apoptotic cell-derived apo-MP have pro-phagocytic activity on AM is still not clear. The aim of this study is to investigate the role of apoptotic ATRA-APL NB4 (ATRA-NB4) cell-derived apo-MP on the phagocytic activity of AM-NR8383 cells in the cell–cell interaction between apoptotic ATRA-NB4 cells and NR8383 cells, this will help us to understand the comprehensive role of CX3CL1(+)apo-MP in the cell–cell interaction between apoptotic APL cells and AM during the resolution phase of acute lung injury in APL patients with DS.

## 2. Materials and Methods

### 2.1. Cell Culture and the Preparation of Conditioned Medium (CM)

NB4 cells (a gift from Dr. M. Lanotte [26]) and NR8383 cells (CRL−2192; ATTC, Manassas, VA, USA) were cultured in RPMI−1640 medium (GIBCO, Grand Island, NY, USA) and in Ham’s F12 K medium, respectively, as described previously [25]. We treated NB4 cells (1 × 10^5^ cells/mL) with ATRA (1 µM; Sigma, St. Louis, MO, USA) for 3–5 days before further studies.

### 2.2. Preparation of Apoptotic Cells

To induce apoptosis, ATRA-NB4 cells were treated with idarubicin (Ida; Pfizer, Milano, Italy) at 5–50 nM/mL for 4 h and incubated at 37 °C [27]. Thereafter, the washed, Ida-treated ATRA-NB4 (Ida-ATRA-NB4) cells were incubated with both annexin V (NXPE; R&D Systems, Minneapolis, MN, USA) and 7-amino-Actinomycin-D (7-AAD; BD Bioscience Pharmingen, San Diego, CA, USA) for double staining before analysis by flowcytometry to measure the percentage of early apoptotic cells and late apoptotic cells, respectively [28].

### 2.3. MP Preparation and Flow Cytometry Analysis

We harvested MP from ATRA-NB4 cell cultures as reported by Gasser et al. and Tsai et al. [29,30]. The MP were stained with annexin V (NXPE; R&D Systems, Minneapolis, MN, USA) or anti-CX3CL1 (MAB365; R&D Systems, Minneapolis, MN, USA) antibodies before flow cytometry analysis.

### 2.4. Assess the Phagocytic Engulfment of Apoptotic Cells by Flowcytometric Analysis

NR8383 cells were first treated with one of the following: CX3CL1 protein (R&D Systems, Minneapolis, MN, USA), anti-CX3CR1 antibody (R&D Systems, Minneapolis, MN, USA), MFG-E8 protein (R&D Systems, Minneapolis, MN, USA), Bay11–7082 (InvivoGen, San Diego, CA, USA) or UO126 (Tocris Bioscience, Bristol, UK). Thereafter, NR8383 cells were washed with 5% PBS solution twice. Subsequently, the NR8383 cells were incubated with PKH−26 (Sigma-Aldrich, St. Louis, MO, USA) labeled Ida-ATRA-NB4 cells (2 × 10^6^) for 30 min before determining the amount of phagocytosis using a BD FACScan [24]. A subset of the Ida-ATRA-NB4 cells was treated with anti-CX3CL1 (R&D Systems, Minneapolis, MN, USA) before labeling with PKH−26. The results were expressed either as the percentage (%) of NR8383 cells with phagocytic activity in engulfing apoptotic cells or as a phagocytosis index that indicates a fold increase relative to the phagocytic activity of untreated NR8383 cells.

### 2.5. Flow Cytometry Analysis of MFG-E8 Expression on NR8383 Cells

Non-permeabilized NR8383 cells were stained with anti-MFG-E8 (Abcam, Cambridge, MA, USA) before analysis by FAC Scan [24].

### 2.6. Measurement of MFG-E8

The level of MFG-E8 in the CM was measured by using an enzyme-linked immunosorbent assay (ELISA) kit (R&D Systems; Minneapolis, MN, USA).

### 2.7. Statistical Analysis

The results were evaluated by the Kolmogorov Smirnov test and/or the Shapiro Wilk test for normal distribution. Thereafter, the results were evaluated by one-way ANOVA followed by the Fisher’s least significant difference (LSD) procedure where appropriate. A value of *p* < 0.05 was considered significant. All data were analyzed by using SPSS software version 23 (IBM SPSS Statistics, Armonk, NY, USA). All results are presented as mean ± SD.

## 3. Results

### 3.1. Apoptotic Cell-Derived MP Have Significant Pro-Phagocytic Activity on NR8383 Cells

We first determined the effect of idarubicin on the induction of apoptosis in ATRA-NB4 cells. Table 1 demonstrates that treating ATRA-NB4 cells with idarubicin for 4 h was able to induce cells to enter the process of apoptosis, during which early apoptotic cells were found to be increased in a dose-dependent manner (*p* < 0.001), while the number of late apoptotic cells showed no significant change.

**Figure 1:**
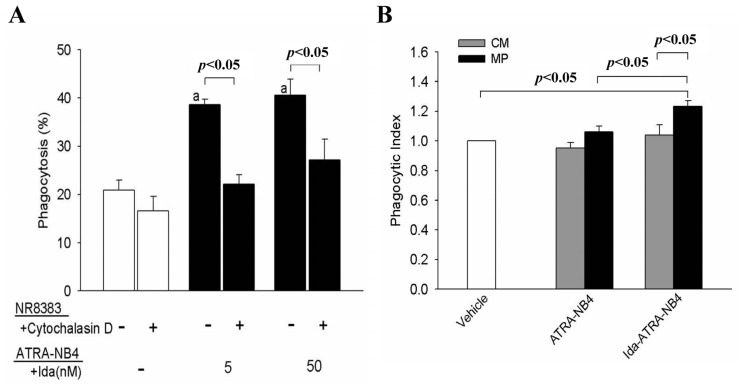
Apoptotic cell-derived MP enhance the phagocytic activity of NR8383 cells. (**A**) NR8383 cells were incubated with either Ida-untreated (blank bar) or Ida-treated (black bar) ATRA-NB4 cells, in the absence or presence of cytochalasin D, before the phagocytic assay. The results are expressed as the percentage of NR8383 cells engulfing ATRA-NB4 cells. The results are the means ± SD from four independent experiments. a: *p* < 0.05 vs. NR8383 cells engulfing Ida-untreated ATRA-NB4 cells in the absence of cytochalasin D treatment (left first blank bar). (**B**) NR8383 cells were pre-incubated with vehicle alone (blank bar), CM (grey bar), or MP (black bar) before phagocytic assay. The results were expressed as a phagocytosis index indicating a fold increase relative to the phagocytic activity of NR8383 cells treated with vehicle alone (blank bar). The results are the means ± SD from five independent experiments.

We recently reported that NR8383 cells can engulf apoptotic ATRA-NB4 cells, as determined by fluorescent microscopic examination [24,31]. In this study, we used flow cytometric analysis to measure the phagocytosis activity of NR8383 cells. Figure 1A demonstrates that the phagocytic activity of NR8383 cells was significantly higher when pre-incubating with apoptotic idarubicin-treated ATRA-NB4 (Ida-ATRA-NB4) cells than that when pre-incubating idarubicin-untreated ATRA-NB4 cells. In addition, the phagocytic activity of NR8383 cells when engulfing apoptotic Ida-ATRA-NB4 cells was significantly inhibited by pre-treatment with cytochalasin D, which is an inhibitor of phagocytosis (Figure 1A); however, this inhibition of phagocytic activity was not observed when NR8383 cells were incubated with idarubicin-untreated ATRA-NB4 cells. This implies that the phagocytic activity of NR8383 cells is enhanced when they are pre-incubated with apoptotic cells but is not enhanced when they are pre-incubated with viable ones. Thereafter, we harvested the MP from either the CM of apoptotic Ida-ATRA-NB4 cell cultures or from the CM of viable ATRA-NB4 cells culture to investigate their role in the phagocytic activity of NR8383 cells. Figure 1B demonstrates that the phagocytic activity of NR8383 cells pre-incubated with apo-MP derived from apoptotic Ida-ATRA-NB4 cells was significantly higher than that of NR8383 cells pre-incubated with vehicle alone, the CM of apoptotic Ida-ATRA-NB4 cell culture or MP harvested from viable ATRA-NB4 cells (*p* < 0.05, *p* < 0.05 and *p* < 0.05; respectively; Figure 1B). However, as compared with NR8383 cells treated with vehicle alone, neither the CM from apoptotic Ida-ATRA-NB4 cells culture nor from viable ATRA-NB4 cells was able to enhance the phagocytosis activity of NR8383 cells. This implies that apoptotic Ida-ATRA-NB4 cell-derived apo-MP possess pro-phagocytic activity on NR8383 cells.

### 3.2. Apoptotic Cell-Derived CX3CL1(+) MP Enhance Phagocytic Activity of NR8383 Cells

**Figure 2:**
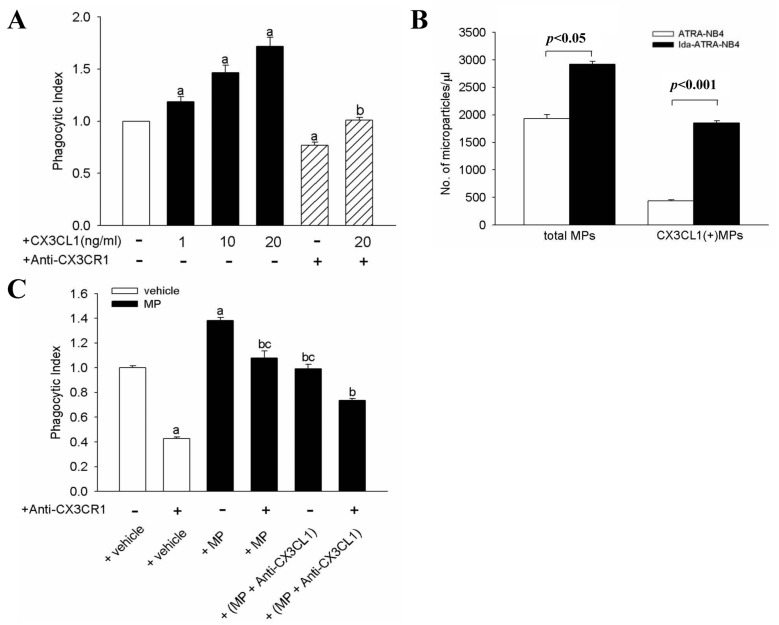
Apoptotic cell-derived CX3CL1(+) MP enhance phagocytic activity of NR8383 cells. (**A**) NR8383 cells were treated with exogenous CX3CL1 (1–20 ng/mL; black bar) before incubation with apoptotic cells for the phagocytic assay. A subset of the NR8383 cells was treated with anti-CX3CR1 antibody before exogenous CX3CL1 treatment (striped bar). The results are expressed as a phagocytosis index, which indicates a fold increase relative to the phagocytic activity of untreated NR8383 cells (blank bar). The results are the means ± SD from five independent experiments a: *p <* 0.05 vs. blank bar; b: *p <* 0.05 vs. NR8383 cells treated with CX3CL1 20 ng/mL only (first right black bar). (**B**) Flow cytometric analysis of MP harvested from the CM of either ATRA-NB4 cell cultures (blank bar) or Idarubicin-treated-ATRA-NB4 cell cultures (black bar). The expression of CX3CL1 and annexin V on the MP was determined by flow cytometric analysis. The total numbers of MP and CX3CL1(+) MP in the 20 mL of CM were calculated using a known concentration of flow count beads. These represent the means+ SD from four independent experiments. (**C**) NR8383 cells were pre-incubated with either vehicle alone (blank bar) or MP (black bar) before the phagocytic assay. Part of the MP was pre-treated with anti-CX3CL1 antibody (MP + Anti-CX3CL1) before incubation with the NR8383 cells. The results were expressed as a phagocytosis index, which indicates a fold increase relative to the phagocytic activity of NR8383 cells treated with vehicle alone (left first blank bar). The results are the means ± SD from four independent experiments. a: *p <* 0.05 vs. left first blank bar; b: *p* < 0.001 vs. left first black bar; c: *p* < 0.001 vs. right first black bar.

We further investigated the role of the CX3CL1–CX3CR1 axis in apo-MP enhanced phagocytic activity in the cell–cell interaction between apoptotic ATRA-NB4 cells and NR8383 cells. We first determined the role of exogenous CX3CL1 in the phagocytic activity of NR8383. Figure 2A demonstrates that exogenous CX3CL1 was able to enhance the phagocytic activity of NR8383 cells in a dose-dependent manner (*p <* 0.05), and that this enhancement was significantly inhibited when NR8383 cells were pre-treated with an antibody specific for CX3CR1 before the exogenous CX3CL1 treatment (*p <* 0.05). Thereafter, we harvested the MP from the CM of ATRA-NB4 cells culture mediums. Figure 2B demonstrates that the numbers of MP and CX3CL1(+) MP were both significantly higher in CM derived from an Ida-ATRA-NB4 cell culture than in CM derived from ATRA-NB4 cell culture (*p <* 0.05 and *p <* 0.001, respectively), implying that increased quantities of MP and CX3CL1(+) MP were released by apoptotic Ida-ATRA-NB4 cells. This is in agreement with our previous study in which ATRA-NB4 cells were induced into apoptosis by UV irradiation [25]. Further study indicates that apo-MP derived from apoptotic Ida-ATRA-NB4 cells can significantly enhance the phagocytic activity of NR8383 cells, as compared with vehicle, when incubated with NR8383 cells (*p* < 0.05, Figure 2C). To further support that CX3CL1–CXCR1 axis contributes to the pro-phagocytic properties of apo-MP derived from apoptotic Ida-ATRA-NB4 cells, Figure 2C demonstrates that the enhanced phagocytic activity of NR8383 cells by apo-MP derived from apoptotic Ida-ATRA-NB4 cells was significantly inhibited by either pre-treating the apo-MP with an anti-CX3CL1 antibody or by pre-treating the NR8383 cells with an anti-CX3CR1 antibody before the phagocytic assay was carried out (*p* < 0.001 and *p* < 0.001, respectively). In addition, this phagocytic activity was further inhibited when NR8383 cells and apo-MP were both pre-treated with antibodies specifically for CX3CR1 and CX3CL1, respectively, before the phagocytic assay was carried out. Taken together, our data indicate that CX3CL1(+) apo-MP released by the apoptotic ATRA-NB4 cells can enhance the phagocytosis activity of NR8383 cells in a CX3CL1–CX3CR1 dependent manner.

### 3.3. Surface CX3CL1 on Ida-ATRA-NB4 Cells Enhances NR8383 Cells’ Phagocytic Activity

**Figure 3:**
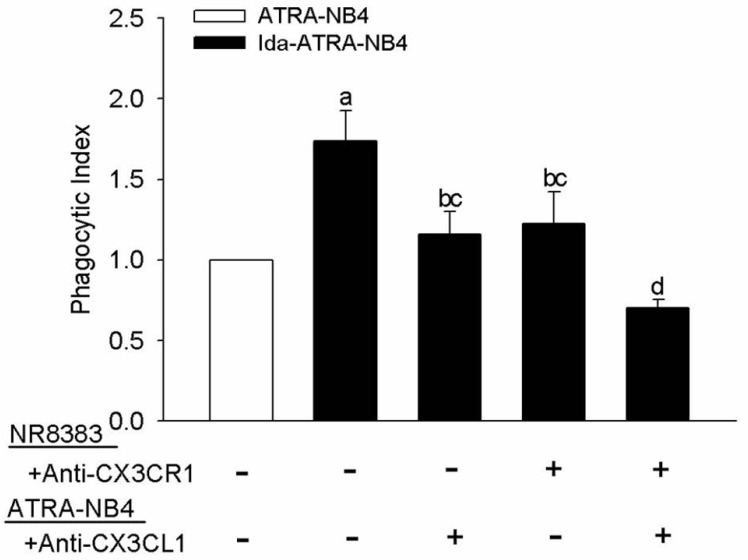
Surface CX3CL1 of apoptotic ATRA-NB4 cells enhances the phagocytic activity of NR8383 cells. NR8383 cells were treated with or without anti-CX3CR1 antibody before incubation with either living cells (blank bar) or apoptotic ATRA-NB4 cells (black bar) for the phagocytic assay. A subset of the Ida-ATRA-NB4 cells was pre-treated with anti-CX3CL1 antibody before incubation with NR8383 cells for the phagocytic assay. The results were expressed as a phagocytosis index, which indicates a fold increase relative to the phagocytic activity of Ida-untreated NR8383 cells (blank bar). The results are the means ± SD from four independent experiments a: *p <* 0.01 vs. blank bar; b: *p <* 0.05 vs. first left black bar; c: *p <* 0.05 vs. first right black bar; d: *p* < 0.001 vs. first left black bar.

We further determined the pro-phagocytic activity of surface CX3CL1 of Ida-ATRA-NB4 cells on NR8383 cells. Figure 3 demonstrates that the phagocytic activity of NR8383 cells, namely the engulfing of apoptotic Ida-ATRA-NB4 cells, was significantly inhibited by either pre-treating the NR8383 cells with anti-CX3CR1 antibody or by pre-treating the apoptotic cells with anti-CX3CL1 antibody before these two cell lines were incubated together for the phagocytic assay (*p <* 0.05 and *p <* 0.05, respectively). This inhibition was further enhanced when the above two antibody-treated cells were incubated together for the phagocytic assay. This implies that CX3CL1 on the surface of apoptotic cells also contribute to the promotion of the phagocytic activity of NR8383 cells in engulfing apoptotic cells on a direct cell–cell contact model.

### 3.4. CX3CL1 and Apoptotic Cell-Derived CX3CL1(+) MP Enhances NR8383 Cells in Surface Expression and Release of MFG-E8

**Figure 4:**
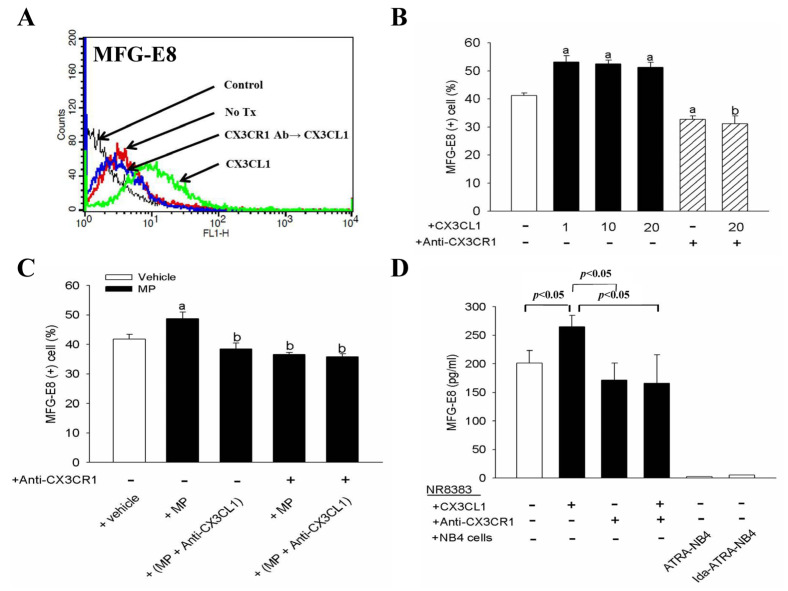
CX3CL1 and apoptotic cell-derived MP enhance NR8383 cells in surface expression and release of MFG-E8. Surface expression of MFG-E8 on NR8383 cells as determined by flow cytometric analysis. (**A**,**B**) NR8383 cells were treated with exogenous CX3CL1 (**B**: black bar) or pre-treated with anti-CX3CR1 antibody before CX3CL1 treatment (**A**: CX3CR1 Ab→CX3CL1; **B**: striped bar). a; *p* < 0.05 vs. blank bar; b; *p* < 0.05 vs. first right black bar. (**A**) This is a representative picture from five independent experiments and (**B**) presents the bar graph data of those five experiments. (**C**) NR8383 cells were incubated with either vehicle alone (blank bar) or Ida-ATRA-NB4 cell-derived apo-MP (black bar); in addition, a subset of the NR8383 cells was pre-treated with anti-CX3CR1 antibody and some of the MP were pre-treated with anti-CX3CL1 antibody (MP + Anti-CX3CL1) before both were incubated together for flow cytometric analysis. a: *p* < 0.05 vs. blank bar; b: *p* < 0.05 vs. left first black bar. (**D**) The levels of MFG-E8 were determined by ELISA in (1) the CM of NR8383 cell culture alone (blank bar); (2) the CM of NR8383 cells treated either CX3CL1, anti-CX3CR1 antibody, or both (black bars); and (3) the CM of NR8383 cells co-cultured with either ATRA-NB4 cells or Ida-ATRA-NB4 cells. (**C**,**D**) The results are the means ± SD from five independent experiments.

In order to determine the role of MFG-E8 on the pro-phagocytic activity of CX3CL1 on NR8383 cells, we investigated the effect of CX3CL1 on the surface expression of MFG-E8 on NR8383 cells and the level of MFG-E8 in the CM of the NR8383 cell cultures. As shown in Figure 4, flow cytometric analysis demonstrated that MFG-E8 was constitutively expressed by NR8383 cells, and its expression was significantly enhanced in NR8383 cells that had been pre-treated with exogenous CX3CL1 (*p <* 0.05). Although no significant dosage effect was observed, this enhancement on MFG-E8 expression was significantly inhibited when NR8383 cells were pre-treated with an anti-CX3CR1 antibody before the exogenous CX3CL1 treatment (Figure 4B). Similarly, the expression of MFG-E8 was also significantly enhanced in NR8383 cells after these cells were incubated with apoptotic Ida-ATRA-NB4 cell-derived apo-MP (*p <* 0.05; Figure 4C), and this enhancement was significantly inhibited either by pre-treating the apo-MP with an anti-CX3CL1 antibody (*p <* 0.05; Figure 4C) or by pre-treating the NR8383 cells with an antibody specific to CX3CR1 before incubation for the phagocytic assay (*p <* 0.05; Figure 4C). After this, we harvested CM from NR8383 cell cultures in order to determine their level of MFG-E8 by ELISA. Figure 4D demonstrates that MFG-E8 was constitutionally released by NR8383 cells, and its level was elevated significantly when NR8383 cells were treated with exogenous CX3CL1; this enhancement was significantly attenuated when NR8383 cells were pre-treated with an antibody specific to CX3CR1 before exogenous CX3CL1 treatment (*p <* 0.05). However, the level of MFG-E8 was markedly decreased in the CM of NR8383 cells co-cultured with either apoptotic Ida-ATRA-NB4 cells or ATRA-NB4 cells (Figure 4D).

### 3.5. MFG-E8 Contributes to the CX3CL1-Enhanced Phagocytic Activity of NR8383 Cells

**Figure 5:**
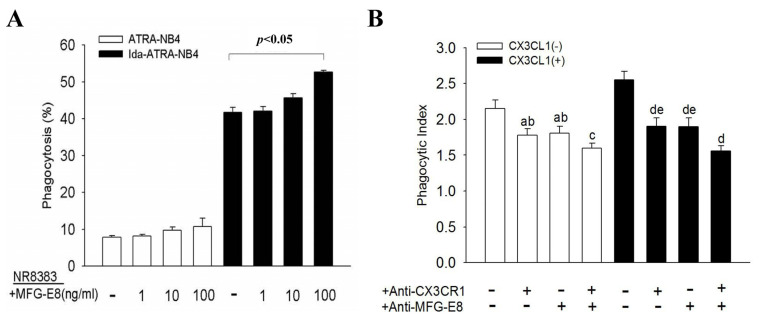
MFG-E8 contributes to the CX3CL1-enhanced phagocytic activity of NR8383 cells. (**A**) NR8383 cells were pre-treated with exogenous MFG-E8 (1–100 ng/mL) before being incubated with either ATRA-NB4 cells (blank bar) or Ida-ATRA-NB4 cells (black bar); they were then subjected to the phagocytic assay. The results are expressed as percentage of NR8383 cells found to be engulfing cells. (**B**) NR8383 cells were first treated with either anti-CX3CR1 antibody or/and anti-MFG-E8 antibody, which was followed by either CX3CL1 treatment (black bar) or no treatment (blank bar) before incubation with apoptotic cells for the phagocytic assay. a: *p <* 0.05 vs. first left blank bar; b: *p* < 0.05 vs. first right blank bar; c: *p <* 0.001 vs. first left blank bar; d: *p <* 0.001 vs. first left black bar; e: *p* < 0.05 vs. first right black bar. (**A**,**B**) The results are the means ± SD from five independent experiments.

Figure 5A demonstrates that exogenous MFG-E8 significantly enhanced the phagocytic activity of NR8383 cells during the engulfing of apoptotic ATRA-NB4 cells, and that this occurs in a dose-dependent manner (*p <* 0.05); however, exogenous MFG-E8 induced no significant change on NR8383 cells in the engulfing of viable ATRA-NB4 cells. Figure 5B further demonstrates that the CX3CL1-enhanced phagocytic activity of NR8383 cells was significantly inhibited by pre-treating NR8383 cells with either an anti-MFG-E8 antibody or an anti-CX3CR1 antibody before exogenous CX3CL1 treatment (*p <* 0.05 and *p <* 0.05, respectively), and this inhibition was further enhanced when NR8383 cells were pre-treated with both antibodies.

### 3.6. CX3CL1 Promotes Phagocytic Activity of NR8383 Cells via the NF-Κb Signal Transduction Pathway

**Figure 6:**
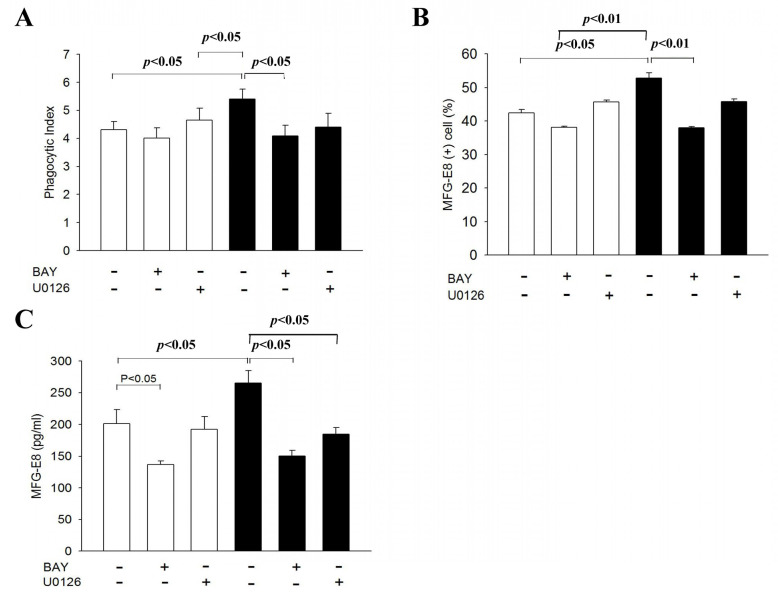
CX3CL1 promotes phagocytic activity of NR8383 cells via the NF-κb signal transducing pathway. (**A**–**C**) NR8383 cells were first treated with either Bay (BAY11–7082; a NF-κb inhibitor) or U0126 (a MARK inhibitor), and this was followed by either exogenous CX3CL1 treatment (black bar) or no treatment (blank bar), before determining (**A**) the phagocytic activity of NR8383 cells when engulfing apoptotic cells; (**B**) the expression of MFG-E8 by flow cytometric analysis; and (**C**) the level of MFG-E8 in the CM of NR8383 cell cultures by ELISA. The results are the means ± SD from four (**A**,**C**) and five (**B**) independent experiments.

Finally, we determined the signal transduction pathway underlying the pro-phagocytic activity of CX3CL1 on NR8383 cells. Figure 6 demonstrates that the phagocytic activity and the MFG-E8 expression of NR8383 cells were both significantly inhibited by pre-treating NR8383 cells with BAY11–7052, an NF-κB inhibitor, before subsequent treatment with CX3CL1 (*p* < 0.05 and *p* < 0.01, respectively); however, this attenuation was not observed in CX3CL1-untreated NR8383 cells. Further studies also demonstrate that the level of MFG-E8 in the CM of NR8383 cells was also significantly inhibited by pre-treatment with Bay11–7082 in NR8383 cells with or without subsequent CX3CL1 treatment (*p* < 0.05 and *p* < 0.05, respectively). In contrast, U0126, an MAPK inhibitor, induced no significant change in the phagocytic activity and did not affect MFG-E8 expression of either CX3CL1-untreated or CX3CL1-treated NR8383 cells, although it did significantly inhibit the level of MFG-E8 in the CM of CX3CL1-treated NR8383 cells.

## 4. Discussion

The results of the present study demonstrate that apoptotic Ida-ATRA-NB4 cell-derived CX3CL1(+) apo-MP promotes the phagocytic activity of NR8383 cells, and enhanced production of MFG-E8 by apo-MP-incubated NR8383 cells is responsible for the enhanced phagocytic activity in the cell–cell interaction between the apoptotic ATRA-NB4 cells and NR8383 cells.

The pro-phagocytic activity of apoptotic ATRA-NB4 cell-derived CX3CL1 on NR8383 cells is strongly supported by the findings that (a) exogenous CX3CL1 promote the phagocytic activity of NR8383 cells in a CX3CR1-dependent manner; (b) apoptotic ATRA-NB4 cell-derived apo-MP promotes the phagocytic activity of NR8383 cells, which was attenuated by pretreatment with CX3CL1- and CX3CR1-specific antibodies, indicating CX3CL1(+) apo-MP possesses pro-phagocytic activity on NR8383 cells; (c) surface CX3CL1 of apoptotic ATRA-NB4 cells promote the phagocytic activity of NR8383 cells in a CX3CR1-dependent manner. Collectively, our results suggest that apoptotic ATRA-NB4 cells have profound effects on the promotion of phagocytic activity of NR8383 cells, and this is through release of CX3CL1 and CX3CL1(+) apo-MP by apoptotic ATRA-NB4 cells as well as through the surface CX3CL1 of the latter cells.

During the process of efferocytic phagocytosis, phosphatidylserine (PS) on the surface of apoptotic cells is the most potent “eat-me” signal, which binds directly or indirectly, via bridging molecules, to phagocytic receptors (TIM−1,4, BAI1, stabilin−2) on the surface of macrophages, and this contributes to the formation of phagocytic synapse between apoptotic cells and macrophages [32]. In this study, we demonstrate that MFG-E8 produced by CX3CL1-treated NR8383 cells is responsible for the pro-phagocytic activity of CX3CL1. To support our proposal, our results demonstrate that (a) CX3CL1 enhances NR8383 cells in their surface expression and release of MFG-E8; (b) the level of MFG-E8 was markedly decreased in the CM of NR8383 cells when the latter cells were incubated with apoptotic ATRA-NB4 cells; (c) CX3CL1-promoted phagocytic activity of NR8383 cells was attenuated when the latter cells were pre-treated with anti-MFG-E8 antibody before performing phagocytic assay; and (d) both enhanced the phagocytic activity and the MFG-E8 expression on CX3CL1-treated NR8383 were attenuated by pre-treating the latter cells with an NF-kB inhibitor before CX3CL1 treatment (Figure 6). Collectively, our results suggest that MFG-E8 produced by CX3CL1-treated NR8383 cells acts as a bridging molecule by potentially promoting the binding between PS on the surface of apoptotic cells and phagocytic receptors (α_v_β_3/5_ integrins) on NR8383 cells, and this contributes to the formation of phagocytic synapses and subsequent activation of the transducing signals for cytoskeletal rearrangement and the engulfment signaling pathway for the efferocytic engulfment of apoptotic ATRA-NB4 cells by NR8383 cells [33,34]. In agreement with our results, previous studies also reported that apoptotic cell-derived CX3CL1 up-regulates *MFG-E8* gene expression and enhances phagocytic activity in microglia [35,36]. The crucial role of CX3CL1 and MFG-E8 during the resolution phase of acute inflammation has been confirmed in previous studies. Miksa et al. also reported that CX3CL1 and MFG-E8 work cooperatively in enhancing the clearance of apoptotic cells by peritoneal macrophages in rats [37]. Impaired clearance of apoptotic cells as well as an excessive infiltration of apoptotic cells in the germinal centers of the spleen have been reported in a MFG-E8 knock-out mice model [38,39]. Parallel to this, we and other groups have reported that annexin I is recruited from the cytosol and exported to the outer plasma membrane leaflet in ATRA-NB4 cells, which co-localizes with PS and also acts as a bridge molecule [30,40]. In addition, DEL−1, Galectin−3 (Gal−3), growth arrest-specific factor 6 (Gas−6), and protein S also act as bridge molecules in the efferocytic process [32]. Further study is warranted to investigate the role other bridge molecules in CX3CL1-enhanced phagocytic activity. Taken together, apoptotic ATRA-NB4 cell-derived CX3CL1 enhance the phagocytic activity of NR8383 cells by up-regulating MFG-E8 as a bridge molecule and contribute to the formation of phagocytic synapses between both cells.

Our results also indicate that the NF-κB signal transduction pathway is involved in the CX3CL1-enhanced MFG-E8 expression and phagocytic activity on NR8383 cells (Figure 6). Sheridan et al. similarly reported that CX3CL1 transduces several well-characterized signaling pathways leading to the activation of NF-κB and the secretion of inflammatory cytokines in microglia [41]. The CX3CL1–CX3CR1 interaction also contributes to the activation of NF-kB through the Akt pathway in an LPS-induced lung injury model [42,43]. The NF-κB signal transduction pathway also mediates the up-regulation of CX3CL1, whereby TNF-α, IL−1β, and lipopolysaccharide up-regulate CX3CL1 in an NF-κB-dependent manner during the inflammatory process [44,45]. However, during the process of efferocytosis, the binding of MFG-E8 with integrin α_v_β_3/5_ in macrophages induces STAT3-mediated SOCS3 activation, leading to the inhibition of the NF-κB signaling pathway and suppression of the production of pro-inflammatory cytokines in LPS-TLR4-mediated inflammatory responses [46,47,48]. Collectively, our results suggest the NF-κB signal transduction pathway plays a crucial role underlying the cooperation of CX3CL1 and MFG-E8 in promoting the phagocytic clearance of apoptotic cells during resolution phase of acute lung injury, and this warrants further studies.

In this study, we address the important role of apo-MP in the cell–cell interaction between apoptotic ATRA-NB4 cells and NR8383 cells during the process of efferocytic phagocytosis. Mohning et al. reported that MP number in the bronchoalveolar lavage fluid was greatest at the peak of inflammation and declined as inflammation resolved during the process of acute lung injury [49]. Soni et al. recently reported that AM were initially the dominant source of MP in the alveolar space during acute lung injury, followed by a large increase in neutrophil-derived MP with the kinetics of neutrophil migration into the alveolar space [50,51]. Our previous study demonstrated that viable ATRA-NB4 cells release annexin A1(+) MP with anti-inflammatory properties by inhibiting the transmigratory and adhesive activity of the recipient cells [30]. In this study, we further demonstrate that apoptotic ATRA-NB4 cells release a large amount of CX3CL1(+) apo-MP to orchestrate NR8383 cells transmigration toward apoptotic cells along a CX3CL1 chemotactic gradient and to condition NR8383 cells with enhanced surface expression of MFG-E8, and this contributes to the formation of phagocytic synapses between the apoptotic ATRA-NB4 cells and N8383 cells before engaging in efferocytic phagocytosis. Additionally, Han et al. reported that AM are able to release anti-inflammatory MP into the alveolar space, and these MP are taken up by alveolar epithelial cells, leading to transcriptional suppression of inflammatory genes in an animal asthma model [52]. Phosphatidylserine on AM-derived MP can even scavenge particulates containing Toll-like receptor ligands or beta-glucans and direct them away from inflammatory and into non-inflammatory compartments [53,54].

In the clinical setting, CX3CL1 and MFG-E8 play an important role during the resolution phase of acute lung injury or sepsis. The lower levels of CX3CL1 and MFG-E8 in the blood, spleen, and liver are associated with an impaired clearance of apoptotic cells and are linked to a poor outcome in septic rats [37,55]. CX3CL1 treatment in animals is able to increase the level of circulating MFG-E8 and prevent tissue injury in a septic mice model, while MFG-E8 treatment of septic rats is also able to attenuate the systemic inflammatory response, enhance the engulfment of apoptotic cells, and improve survival [37,55]. MFG-E8 also shows a direct anti-inflammatory role independent of the phagocytic engulfment of apoptotic cells. In this context, Aziz et al. reported that recombinant MFG-E8 lowered intestinal inflammation by directly regulating TLR4 signaling through its binding with integrin α_v_β_3_ [46]. Therefore, our results provide a rationale for the clinical usage of CX3CL1 and/or MFG-E8 in treating acute lung injury in patients with DS or acute lung injury caused by other pathogens, and this also warrants further clinical studies.

## Figures and Tables

**Table 1:** Idarubicin Induces ATRA-NB4 Cells to Enter the Process of Apoptosis.

Idarubicin (nM)	* Early Apoptosis (%)	* Late Apoptosis (%)
0	11.0 ± 0.6	9.1 ± 0.3
5	21.0 ± 1.0	7.6 ± 1.5
50	45.8 ± 0.4	6.3 ± 0.4
*p* value **	*p* < 0.001	NS

* Early apoptosis is defined as cells expressing annexin V but not 7-AAD; late apoptosis is defined as cells expressing both annexin V and 7-AAD. ** ANOVA. NS: not significant.

## Data Availability

Not applicable.

## References

[1] Frankel S.R., Eardley A., Lauwers G., Weiss M., Warrell R.P. (1992). The “retinoic acid syndrome” in acute promyelocytic leukemia. Ann. Intern. Med..

[2] Montesinos P., Bergua J.M., Vellenga E., Rayón C., Parody R., de la Serna J., León A., Esteve J., Milone G., Debén G. (2009). Differentiation syndrome in patients with acute promyelocytic leukemia treated with all-trans retinoic acid and anthracycline chemotherapy: Characteristics, outcome, and prognostic factors. Blood.

[3] Camacho L.H., Soignet S.L., Chanel S., Ho R., Heller G., Scheinberg D.A., Ellison R., Warrell R.P. (2000). Leukocytosis and the retinoic acid syndrome in patients with acute promyelocytic leukemia treated with arsenic trioxide. J. Clin. Oncol..

[4] Jimenez J.J., Chale R.S., Abad A.C., Schally A.V. (2020). Acute promyelocytic leukemia (APL): A review of the literature. Oncotarget.

[5] Luesink M., Jansen J.H. (2010). Advances in understanding the pulmonary infiltration in acute promyelocytic leukaemia. Br. J. Haematol..

[6] Dubois C., Schlageter M.H., de Gentile A., Guidez F., Balitrand N., Toubert M.E., Krawice I., Fenaux P., Castaigne S., Najean Y. (1994). Hematopoietic growth factor expression and ATRA sensitivity in acute promyelocytic blast cells. Blood.

[7] Sanz M.A., Montesinos P. (2014). How we prevent and treat differentiation syndrome in patients with acute promyelocytic leukemia. Blood.

[8] Seale J., Delva L., Renesto P., Balitrand N., Dombret H., Scrobohaci M.L., Degos L., Paul P., Chomienne C. (1996). All-trans retinoic acid rapidly decreases cathepsin G synthesis and mRNA expression in acute promyelocytic leukemia. Leukemia.

[9] Marchetti M., Falanga A., Giovanelli S., Oldani E., Barbui T. (1996). All-trans-retinoic acid increases adhesion to endothelium of the human promyelocytic leukaemia cell line NB4. Br. J. Haematol..

[10] Robb C., Regan K., Dorward D., Rossi A. (2016). Key Mechanisms Governing Resolution of Lung Inflammation, Seminars in Immunopathology. Semin Immunopathol..

[11] Levy B.D., Clish C.B., Schmidt B., Gronert K., Serhan C.N. (2001). Lipid mediator class switching during acute inflammation: Signals in resolution. Nat. Immunol..

[12] Serhan C., Chiang N. (2008). Endogenous pro-resolving and anti-inflammatory lipid mediators: A new pharmacologic genus. Br. J. Pharmacol..

[13] Buckley C.D., Gilroy D.W., Serhan C.N. (2014). Proresolving lipid mediators and mechanisms in the resolution of acute inflammation. Immunity.

[14] Park S.-Y., Kim I.-S. (2017). Engulfment signals and the phagocytic machinery for apoptotic cell clearance. Exp. Mol. Med..

[15] McCracken J.M., Allen L.-A.H. (2014). Regulation of human neutrophil apoptosis and lifespan in health and disease. J. Cell Death.

[16] Perretti M., D’acquisto F. (2009). Annexin A1 and glucocorticoids as effectors of the resolution of inflammation. Nat. Rev. Immunol..

[17] Serhan C.N., Brain S.D., Buckley C.D., Gilroy D.W., Haslett C., O’Neill L.A., Perretti M., Rossi A.G., Wallace J.L. (2007). Resolution of in flammation: State of the art, definitions and terms. FASEB J..

[18] Barnig C., Bezema T., Calder P.C., Charloux A., Frossard N., Garssen J., Haworth O., Dilevskaya K., Levi-Schaffer F., Lonsdorfer E. (2019). Activation of resolution pathways to prevent and fight chronic inflammation: Lessons from asthma and inflammatory bowel disease. Front. Immunol..

[19] Kourtzelis I., Hajishengallis G., Chavakis T. (2020). Phagocytosis of apoptotic cells in resolution of inflammation. Front. Immunol..

[20] Ravichandran K.S. (2010). Find-me and eat-me signals in apoptotic cell clearance: Progress and conundrums. J. Exp. Med..

[21] Doran A.C., Yurdagul A., Tabas I. (2020). Efferocytosis in health and disease. Nat. Rev. Immunol..

[22] Kourtzelis I., Mitroulis I., von Renesse J., Hajishengallis G., Chavakis T. (2017). From leukocyte recruitment to resolution of inflammation: The cardinal role of integrins. J. Leukoc. Biol..

[23] Bazan J.F., Bacon K.B., Hardiman G., Wang W., Soo K., Rossi D., Greaves D.R., Zlotnik A., Schall T.J. (1997). A new class of membrane-bound chemokine with a CX 3 C motif. Nature.

[24] Tsai W.-H., Shih C.-H., Feng S.-Y., Li I.-T., Chang S.-C., Lin Y.-C., Hsu H.-C. (2014). CX3CL1 (+) microparticles mediate the chemoattraction of alveolar macrophages toward apoptotic acute promyelocytic leukemic cells. Cell. Physiol. Biochem..

[25] Tsai W.-H., Shih C.-H., Feng S.-Y., Chang S.-C., Lin Y.-C., Hsu H.-C. (2014). Role of CX3CL1 in the chemotactic migration of all-trans retinoic acid-treated acute promyelocytic leukemic cells toward apoptotic cells. J. Chin. Med. Assoc..

[26] Lanotte M., Martin-Thouvenin V., Najman S., Balerini P., Valensi F., Berger R. (1991). NB4, a maturation inducible cell line with t (15; 17) marker isolated from a human acute promyelocytic leukemia (M3). Blood.

[27] Liu Y., Chen F., Wang S., Guo X., Shi P., Wang W., Xu B. (2013). Low-dose triptolide in combination with idarubicin induces apoptosis in AML leukemic stem-like KG1 a cell line by modulation of the intrinsic and extrinsic factors. Cell Death Dis..

[28] Hasper H., Weghorst R., Richel D., Meerwaldt J., Olthuis F., Schenkeveld C. (2000). A new four-color flow cytometric assay to detect apoptosis in lymphocyte subsets of cultured peripheral blood cells. Cytom. J. Int. Soc. Anal. Cytol..

[29] Gasser O., Hess C., Miot S., Deon C., Sanchez J.-C. (2003). Characterisation and properties of ectosomes released by human polymorphonuclear neutrophils. Exp. Cell Res..

[30] Tsai W.H., Chien H.Y., Shih C.H., Lai S.L., Li I.T., Hsu S.C., Kou Y.R., Hsu H.C. (2012). Annexin A1 mediates the anti-inflammatory effects during the granulocytic differentiation process in all-trans retinoic acid-treated acute promyelocytic leukemic cells. J. Cell. Physiol..

[31] Tsai W.H., Shih C.H., Feng S.Y., Li I.T., Chang S.C., Lin Y.C., Hsu H.C. (2014). CX3CL1(+) Microparticles Mediate the Chemoattraction of Alveolar Macrophages toward Apoptotic Acute Promyelocytic Leukemic Cells. Cell. Physiol. Biochem. Int. J. Exp. Cell. Physiol. Biochem. Pharmacol..

[32] Elliott M.R., Ravichandran K.S. (2016). The dynamics of apoptotic cell clearance. Dev. Cell.

[33] Lemke G. (2019). How macrophages deal with death. Nat. Rev. Immunol..

[34] Akakura S., Singh S., Spataro M., Akakura R., Kim J.-I., Albert M.L., Birge R.B. (2004). The opsonin MFG-E8 is a ligand for the αvβ5 integrin and triggers DOCK180-dependent Rac1 activation for the phagocytosis of apoptotic cells. Exp. cell Res..

[35] Leonardi-Essmann F., Emig M., Kitamura Y., Spanagel R., Gebicke-Haerter P.J. (2005). Fractalkine-upregulated milk-fat globule EGF factor−8 protein in cultured rat microglia. J. Neuroimmunol..

[36] Fuller A.D., Van Eldik L.J. (2008). MFG-E8 regulates microglial phagocytosis of apoptotic neurons. J. Neuroimmune Pharmacol..

[37] Miksa M., Amin D., Wu R., Dong W., Ravikumar T.S., Wang P. (2007). Fractalkine-induced MFG-E8 leads to enhanced apoptotic cell clearance by macrophages. Mol. Med..

[38] Hanayama R., Tanaka M., Miyasaka K., Aozasa K., Koike M., Uchiyama Y., Nagata S. (2004). Autoimmune disease and impaired uptake of apoptotic cells in MFG-E8-deficient mice. Science.

[39] Asano K., Miwa M., Miwa K., Hanayama R., Nagase H., Nagata S., Tanaka M. (2004). Masking of phosphatidylserine inhibits apoptotic cell engulfment and induces autoantibody production in mice. J. Exp. Med..

[40] Arur S., Uche U.E., Rezaul K., Fong M., Scranton V., Cowan A.E., Mohler W., Han D.K. (2003). Annexin I is an endogenous ligand that mediates apoptotic cell engulfment. Dev. Cell.

[41] Sheridan G.K., Murphy K.J. (2013). Neuron–glia crosstalk in health and disease: Fractalkine and CX3CR1 take centre stage. Open Biol..

[42] Ding X.-M., Pan L., Wang Y., Xu Q.-Z. (2016). Baicalin exerts protective effects against lipopolysaccharide-induced acute lung injury by regulating the crosstalk between the CX3CL1-CX3CR1 axis and NF-κB pathway in CX3CL1-knockout mice. Int. J. Mol. Med..

[43] Meucci O., Fatatis A., Simen A.A., Miller R.J. (2000). Expression of CX3CR1 chemokine receptors on neurons and their role in neuronal survival. Proc. Natl. Acad. Sci. USA.

[44] Moon S.O., Kim W., Sung M.J., Lee S., Kang K.P., Kim D.H., Lee S.Y., So J.N., Park S.K. (2006). Resveratrol suppresses tumor necrosis factor-alpha-induced fractalkine expression in endothelial cells. Mol. Pharmacol..

[45] Garcia G.E., Xia Y., Chen S., Wang Y., Ye R.D., Harrison J.K., Bacon K.B., Zerwes H.G., Feng L. (2000). NF-κB-dependent fractalkine induction in rat aortic endothelial cells stimulated by IL−1β, TNF-α, and LPS. J. Leukoc. Biol..

[46] Aziz M.M., Ishihara S., Mishima Y., Oshima N., Moriyama I., Yuki T., Kadowaki Y., Rumi M.A.K., Amano Y., Kinoshita Y. (2009). MFG-E8 attenuates intestinal inflammation in murine experimental colitis by modulating osteopontin-dependent αvβ3 integrin signaling. J. Immunol..

[47] Yi Y.-S. (2016). Functional role of milk fat globule-epidermal growth factor VIII in macrophage-mediated inflammatory responses and inflammatory/autoimmune diseases. Mediat. Inflamm..

[48] Miksa M., Amin D., Wu R., Jacob A., Zhou M., Dong W., Yang W.-L., Ravikumar T.S., Wang P. (2008). Maturation-induced down-regulation of MFG-E8 impairs apoptotic cell clearance and enhances endotoxin response. Int. J. Mol. Med..

[49] Mohning M.P., Thomas S.M., Barthel L., Mould K.J., McCubbrey A.L., Frasch S.C., Bratton D.L., Henson P.M., Janssen W.J. (2018). Phagocytosis of microparticles by alveolar macrophages during acute lung injury requires MerTK. Am. J. Physiol.-Lung Cell. Mol. Physiol..

[50] Soni S., Wilson M.R., O’Dea K.P., Yoshida M., Katbeh U., Woods S.J., Takata M. (2016). Alveolar macrophage-derived microvesicles mediate acute lung injury. Thorax.

[51] Reutershan J., Basit A., Galkina E.V., Ley K. (2005). Sequential recruitment of neutrophils into lung and bronchoalveolar lavage fluid in LPS-induced acute lung injury. Am. J. Physiol.-Lung Cell. Mol. Physiol..

[52] Han C.Z., Juncadella I.J., Kinchen J.M., Buckley M.W., Klibanov A.L., Dryden K., Onengut-Gumuscu S., Erdbrügger U., Turner S.D., Shim Y.M. (2016). Macrophages redirect phagocytosis by non-professional phagocytes and influence inflammation. Nature.

[53] Freeman S.A., Grinstein S. (2016). Phagocytosis: How macrophages tune their non-professional counterparts. Curr. Biol..

[54] Stokes C.A., Kaur R., Edwards M.R., Mondhe M., Robinson D., Prestwich E.C., Hume R.D., Marshall C.A., Perrie Y., O’Donnell V.B. (2016). Human rhinovirus-induced inflammatory responses are inhibited by phosphatidylserine containing liposomes. Mucosal Immunol..

[55] Miksa M., Wu R., Dong W., Das P., Yang D., Wang P. (2006). Dendritic cell-derived exosomes containing milk fat globule epidermal growth factor-factor VIII attenuate proinflammatory responses in sepsis. Shock.

